# The role of staphylococci in subclinical mastitis of cows and lytic phage isolation against to *Staphylococcus aureus*

**DOI:** 10.14202/vetworld.2017.1481-1485

**Published:** 2017-12-16

**Authors:** Aliye Gülmez Sağlam, Mitat Şahin, Elif Çelik, Özgür Çelebi, Doğan Akça, Salih Otlu

**Affiliations:** 1 Department of Microbiology, Faculty of Veterinary Medicine, University of Kafkas, Kars, Turkey; 2 Faculty of Health Sciences, University of Kafkas, Kars, Turkey

**Keywords:** bacteriophage, dairy cow, *Staphylococcus aureus*, subclinical mastitis

## Abstract

**Aim:**

This study was conducted to determine the role of *Staphylococcus* in the formation of subclinical mastitis in cows and to isolate the phage against isolated *Staphylococcus aureus* strains.

**Materials and Methods:**

In this study, 400 milk cows were screened by California Mastitis Test (CMT) for subclinical mastitis and 235 udders of 96 cows, which were determined to be positive, were evaluated for *Staphylococcus*. Milk samples were evaluated using conventional and molecular methods. In addition, phage isolation studies were performed against *S. aureus* strains causing mastitis.

**Results:**

At the result of cultural examination, of 235 milk samples that were found as positive for mastitis by CMT, a total of 117 (49.7%) *Staphylococcus* spp. were isolated as a distribution of 74 (63.24%) coagulase-positive staphylococci and 43 (36.75%) coagulase-negative staphylococci. Of these isolates, 76 (64.95%) were characterized as *S. aureus* both conventional and molecular techniques. Lytic bacteriophages against two *S. aureus* strains which were isolated from mastitic milk samples were obtained from wastewater samples.

**Conclusion:**

The results of this study show that a significant portion of subclinical mastitis was formed by staphylococci. In addition, phage isolation against *S. aureus* strains isolated can be considered as one of the steps to be applied in the prophylaxis and treatment of such infections.

## Introduction

Mastitis is an inflammation of mammary gland and characterized by physical, chemical, and bacteriological changes in milk and pathologic changes in the glandular tissue. In this, particular subclinical mastitis is one of the most common forms of disease of highly productive dairy animals [1]. Mastitis has a great economic importance in dairy industry due to the causes as decreasing in milk yield and quality [2]. It has also a serious zoonotic potential due to the distribution of bacteria and toxins through the milk [3].

Mastitis caused by a series of pathogen is classified as contagious and environmental epidemiologically. In dairy cows, more than 140 microorganisms have been found as the cause of mastitis [3,4]. Contagious agents use infected udder as a reservoir, spread from cow to cow during milking, and can transform to chronic subclinical infections with the appearance of clinical cases. *Staphylococcus aureus*, *Streptococcus agalactiae*, *Mycoplasma* spp., and *Corynebacterium bovis* are placed among the contagious pathogens [5].

The most important microorganism causing mastitis is *Staphylococcus* spp., and particularly, *S. aureus* is responsible for about one-third of cases of clinical and subclinical mastitis [4,6]. Staphylococci are cocci which facultative anaerobe, Gram-positive, catalase-positive in the family *Micrococcaceae*. Staphylococci are a part of normal bacteria flora in mammals and birds. In veterinary medicine, *S. aureus* causes mastitis in animal species such as cattle, sheep, goats, and horses; dermatitis in sheep and goats; and botryomycosis in pigs and horses [7].

The reasons for the failure of treatment in *S. aureus*-associated mastitis include the coinfection of several breast lobes, the length of treatment period, and infection occurrence due to *S. aureus* producing beta-lactamase. Furthermore, *S. aureus* forms an abscess surrounded by thick fibrous capsules in the mammary gland. This can prevent the adequate accumulation of antibiotic on target site, and thus, bacterial destroying is interrupted [8,9].

Microorganisms can gain resistance to the antibiotics used in mastitis treatment [10]. Penicillin resistance of *S. aureus* in mastitis cases was reported as 50% in the USA, 71.4% in Ireland, and 67.3% in England [11]. Güler *et al*. [12] reported that 63.3% of *S. aureus* strains were resistant to penicillin and ampicillin in a study conducted in Turkey. As it is understood from the results of the studies made, *S. aureus* can develop high resistance against to commonly used antibiotics. These problems indicate that there is a need for alternative treatment for this bacterial disease [13]. Bacteriophages, defined as bacterial viruses, can be used as antibacterial agents in battle against to bacterial infection. Unlike the antibiotics, phages can specifically kill the target bacteria and do not affect to the normal microflora of the host [10]. This brought the phage therapy attractive against mastitis infections associated with *S. aureus*. Studies have shown that some phages were successfully isolated against *S. aureus* strains causing bovine mastitis [13].

This study was designed to determine the role of *Staphylococcus* in the formation of subclinical mastitis in cows and to isolate and purify phage from various samples against isolated *S. aureus* strains.

## Materials and Methods

### Ethical approval

The experiment was carried out with the approval of the Local Ethical Committee in Kars (KAÜ-HADYEK/2015-024).

### Milk samples

In this study, 400 cows rising in 12 family-type farms in Kars were examined with California Mastitis Test (CMT) for subclinical mastitis. The CMT was performed according to the methodology developed by Schalm *et al*. [14]. A total of 235 milk samples taken from 96 cows determined as positive by CMT were evaluated for *Staphylococcus* spp. Right after the nipples were cleaned with 70% ethyl alcohol, the first milking discharge was collected under aseptic conditions. It was taken approximately 40 ml of milk into sterilized Falcon tube from each udder lobe of animals which were determined to be subclinical mastitis by CMT. Milk samples were transported to Kafkas University, Faculty of Veterinary Medicine, Microbiology Department Laboratories in cold chain and short time.

### Isolation and identification

For isolation of *Staphylococcus* spp., 100 μl of milk samples were homogenized by vortexing and then streaked on to Blood Agar (Oxoid CM0271) enriched with 7% sheep blood and incubated at 37°C for 24-48 h in aerobic condition. After incubation, cultures were evaluated in terms of colony morphology, pigment production, and hemolysis, and Gram-staining features were examined, as well. Suspicious colonies were identified by subjecting to the classical biochemical tests (i.e., catalase, oxidase, urease, and nitrate reduction tests) [15].

### Molecular diagnosis

DNA extraction from *Staphylococcus* spp. isolated from milk samples was performed by the phenol-chloroform method [16]. Molecular typing of isolates was carried out according to the polymerase chain reaction (PCR) method developed by Riffon *et al*. [17]. *S. aureus* reference strain (ATCC 6538) was used as a positive control and ddH_2_O was used as a negative control. In this study, it was used Sau 327 (’-GGACGACATTAGACGAATCA-3’) and Sau 1645 (5’-CGGGCACCTATTTTCTATCT-3’) primers for the 1318 base pairs (bp) length amplification of 23S rRNA gene specific for *S. aureus*. Specific bands were photographed after 1.5% agarose gel electrophoresis of the amplified products.

### Phage isolation

Mastitic milk and farm wastewater samples were used as material, and field *S. aureus* isolates were used as host for lytic phage isolation against *S. aureus*. The method was performed according to the methodology developed by Oliveira *et al*. [18]. Briefly, 10 ml 10× Brain Heart Infusion (BHI) broth and 100 µl bacterial culture obtained by adding of 3 ml of BHI broth onto the 24 h fresh slant agar culture of *S. aureus* were added into the bottles containing 90 ml of mastitic milk or farm wastewater samples and the bottles were incubated at 37°C for 24 h in shaker incubator. After the incubation, suspensions were centrifuged and the supernatants were filtered through a 0.22 μm filter. To determine the phage-host sensitivity, bacterial eluate was prepared by adding 3 ml BHI broth onto the fresh *S. aureus* slant culture and vortexed vigorously. 100 μl of eluate was added onto 3 ml of soft agar and plated on BHI agar plates. 15 μl of suspected phage suspension was dropped on the agar plates containing host bacteria, and the plates were incubated aerobically at 37°C for 24 h. Bacteriophage activity was evaluated by the presence of plaque formation on BHI agar plates.

### Purification of phages

To purify the phage, the double-layer agar method was used [19]. For this purpose, serial dilution of phage was prepared till to 10^−7^. Then, 1 ml sample was taken from each dilution and mixed with 100 μl fresh target bacterial culture, and 3 ml of soft agar was added thereto. The mixture was spreaded on BHI agar plates and incubated at 37°C for 24 h. Different size phage plaques on agar plates were observed and picked up and transferred separately onto the 1 ml of BHI broth by sterile pipet tips and vortexed thoroughly by adding 1 ml BHI broth. The double-layer agar method was repeated for at least 5 times until a uniform plaque was formed.

## Results

### Isolation and identification of *S. aureus*

**Figure-1:**
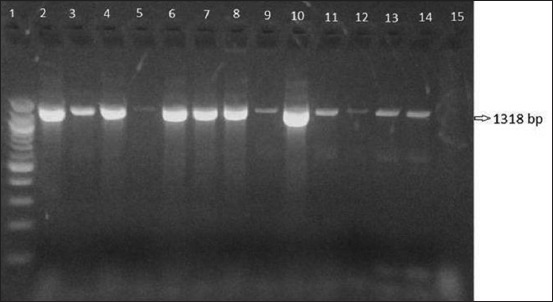
The image of gel electrophoresis of *Staphylococcus aureus*-specific polymerase chain reaction. 1: DNA ladder (Gene ruler 100 bp DNA ladder plus, Fermentas), 2: Positive control (ATCC 6538), 3-14: Positive samples, 15: Negative control (ddH_2_O).

As the results of cultural examination, of 235 milk samples that were found mastitic by CMT, a total of 117 (49.7%) *Staphylococcus* spp. were isolated as a distribution of 74 (63.24%) coagulase-positive staphylococci (CPS) and 43 (36.75%) coagulase-negative staphylococci (CNS). Of these isolates, 76 (64.95%) were characterized as *S. aureus* both conventional and molecular techniques (Figure-1). Among the *S. aureus* isolates, 7 were found having coagulase-negative activity. Apart from these, the 5 isolates identified as CNS by conventional tests were found not to be *S. aureus* by PCR, as well.

### Phage isolation and purification

**Figure-2:**
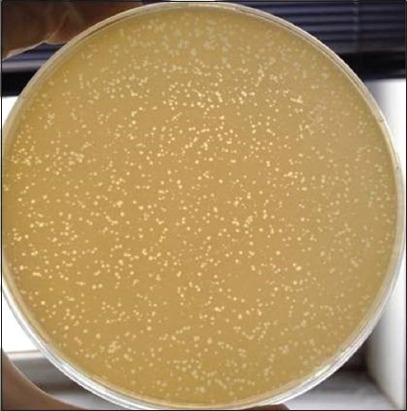
Demonstration of phage presence by double agar method.

While the phage isolation from mastitic milk samples was not achieved, using the wastewater samples, phage was isolated against to two *S. aureus* strains within 35 strains selected randomly among *S. aureus* strains isolated from mastitic milk samples. These phages were found having lytic activity on *S. aureus* field isolates and then purified and concentrated for future uses (Figure-2).

## Discussion

Bovine mastitis is an economically important disease of the dairy industry worldwide. Although various pathogens are listed, *Staphylococcus* spp., in particular *S. aureus*, is considered as one of the most important agents of clinical and subclinical mastitis. The cases caused by the antibiotic-resistant variants (methicillin-resistant *S. aureus* [MRSA] or vancomycin-resistant *S. aureus*) of *S. aureus* are more important due to the tendency to replicate chronically [20]. In a study conducted in Sweden, 31% *S. aureus* and 27% CNS were isolated at the result of cultural examination of 583 milk samples from subclinical mastitic cow in 226 different farms [21]. In a different study in Ireland, samples taken from infected breast lobes of 285 cattle from 15 different farms were evaluated and 61 (21%) *S. aureus* and 26 (9%) CNS were reported [22]. Tel *et al*. [23] isolated 84 (32.5%) *S. aureus* and 71 (27.5%) CNS from 258 milk samples in cattle in a study in Şanlıurfa region. In a study conducted by Sevinti and Şahin [24] in Kars Province, 23 (34.3%) *S. aureus* and 19 (28.3%) CNS were isolated from 79 cow milk samples from mastitic breast lobes. In the present study, 117 (49.7%) *Staphylococcus* spp. were isolated from 235 cow milk samples with subclinical mastitis. Of 117 isolates, 74 (63.24%) and 43 (36.75%) were determined as CPS and CNS, respectively. By species-specific PCR, 76 (64.95%) of these isolates were identified as *S. aureus*. Among the *S. aureus* isolates, 7 were found having coagulase-negative activity. Apart from these, the 5 isolates identified as CNS by conventional tests were found not to be *S. aureus* at the result of species-specific PCR, as well. In this study, *S. aureus* is the bacterium which was isolated at the highest rate among the bacterial agents causing mastitis, as well. This seems a good harmony with the other studies’ isolation results.

Bacteriophages have a significant potential as an antibacterial agent. Bacteriophage can be used in animal husbandry as an additive therapy besides of infection management of antibiotic-resistant *Staphylococcus* spp. Slanetz and Jawetz [25] showed the presence of lytic staphylococcal bacteriophages from mastitic cow milks but suggested that these phages were not freely in milk, always linked to some particles. Gill *et al*. [26] have identified those proteins which are carriers of phages in whey. Researchers showed that these proteins inhibit phage binding to the cell surfaces and thus prevent the phage adsorption and cell lysis. While specific bacteriophages are isolated against *S. aureus*, make thought that their lytic activities and *in vitro* conditions can be used to control of many pathological events. In a study, it was showed that phage efficiency is insufficient against subclinical mastitis caused by *S. aureus* [27]. In another study, Gill *et al*. [26] used 18 breast lobes with mastitis caused by *S. aureus* of 13 cows. Ten ml bacteriophage with a concentration of 1.25×10^10^ pfu/ml were given into infected breast lobes for over 5 days, and no pathogen presence was determined in 3 of 18 breast lobes. This ratio was not statistically significant, and thus, it was suggested that phages may be inactive in the mammary gland. Recently, Kwiatek *et al*. [10] have isolated lytic bacteriophages against *S. aureus* from the milk through standard enrichment using a mixed culture of randomly selected *S. aureus* ATCC 43300 and ATCC 25923 and *S. aureus* MRSA strains.

O’Flaherty *et al*. [28] gave three phage cocktails at 10^8^ pfu/ml concentration into the cow’s milk and showed that it could not provide detectably increase in the number of somatic cell counts indicating an immune response for a high number of phages. In an *in vivo* study conducted by Wills *et al*. [29], it was showed that the numbers of phages in treated animals were higher than initially given, suggested that phage proliferation was successfully performed in animal tissues. Capparelli *et al*. [30] tested a phage against *S. aureus* in an experimental study in mice and showed that 97% of the infected animals achieved success as a result of the study over 10 days. In the current study, bacteriophage isolation from mastitic milks against *S. aureus* strains isolated from mastitic cattle milks could not be achieved, but it was successfully carried out from waste farm waters. Phages were isolated and purified using double-layer agar method and showed lytic activity *in vitro* conditions against *S. aureus* strains isolated from mastitic cattle milks. These purified phages need to be titrated and some experimental studies to determine their effect on mastitis are required, and quite frankly, it is not considered in this research plan, but it is thought that this could be a separate study topic.

## Conclusion

The results of this study reveal that a significant (49.7%) proportion of subclinical mastitis of the cows was formed by staphylococci and that *S. aureus* was the predominant (64.95%) species. In addition, lytic phage isolation from farm wastewater samples specific to *S. aureus* can be considered as a preliminary step to investigate its usefulness in the prophylaxis and treatment of such infections.

## Authors’ Contributions

AGS carried out the study. SO and MŞ planned, designed, and supervised the experiment. EÇ, ÖÇ, and DA carried out the sampling and species identification. All authors read and approved the manuscript.
